# COVID-19 Induces Body Composition and Metabolic Alterations

**DOI:** 10.7759/cureus.34196

**Published:** 2023-01-25

**Authors:** Mohammad Hosein Yazdanpanah, Maryam Mardani, Saeed Osati, Elham Ehrampoush, Seyed Hossein Davoodi, Reza Homayounfar

**Affiliations:** 1 Student Research Committee, Fasa University of Medical Sciences, Fasa, IRN; 2 Student Research Committee, Shiraz University of Medical Sciences, Shiraz, IRN; 3 Faculty of Nutrition and Food Technology, National Nutrition and Food Technology Research Institute, Shahid Beheshti University of Medical Sciences, Tehran, IRN

**Keywords:** muscle loss fat gain, bmi, coronavirus disease 2019, fat distribution, endocrinology, body composition, metabolism, covid-19

## Abstract

Background

Individuals with severe acute respiratory syndrome coronavirus 2 (SARS-CoV-2) infection are highly susceptible to disease-related metabolic dysregulation given the hyperinflammatory immune response of coronavirus disease 2019 (COVID-19). These changes are remarkably involved in multiple steps in adipogenesis and lipolysis. This study aimed to elaborate on the significant relations of COVID-19 infection with body fat distribution, changes in serum insulin, and homeostasis model assessment-estimated insulin resistance (HOMA-IR) levels before and after the infection.

Methods

In this follow-up study, from July 2021 to September 2021, persons referred to a university-affiliated Nutrition Counselling Clinic were randomly selected for the study samples. Validated food frequency (FFQ) and physical activity questionnaires were completed. Body composition was assessed in this investigation. On the second visit, those who reported mild to moderate COVID-19 infection (without hospitalization) were selected as the case group and the asymptomatic individuals as the control group. All measurements were re-measured in the second visit.

Results

In a total of 441 patients, the mean age was 38.82±4.63 years. There were 224 (50.79%) male subjects, and 217 (49.20%) were females. There was a statistically significant difference in the longitudinal change in total fat percentage between subjects with and without COVID-19. Also, the difference in HOMA-IR before and after COVID-19 in case groups (both males and females) was statistically significant (P-value < 0.001). Moreover, serum insulin levels were significantly increased in all cases (P-value < 0.001), while remaining stable in control groups. When compared to their initial visit, COVID-19 patients' total fat percentage rose significantly (almost 2%) following a hypocaloric diet. Participants who were not infected with COVID-19 had a lower total fat percentage than those who were. Serum insulin and HOMA-IR levels increased significantly after infection compared to the primary measurements.

Conclusion

Individuals with COVID-19 infection may require tailored medical nutrition therapy to improve short and long-term COVID-19 outcomes such as muscle loss and fat accommodation.

## Introduction

Severe acute respiratory syndrome coronavirus 2 (SARS-CoV-2), caused by the novel coronavirus, has rapidly caused a global health crisis. Coronavirus disease 2019 (COVID-19) clinical manifestations range from asymptomatic individuals to respiratory arrest needing mechanical ventilation, with a significant death and morbidity rate, especially in the elderly [1]. In the pathophysiology of COVID-19 infection, the angiotensin-converting-enzyme receptor 2 (ACE2) plays a critical role [2]. It interacts with SARS-CoV-2 spike protein S, facilitating virion attachment and entry into the host genome [3,4]. Following the entrance, the virus induces severe cell damage through the hyperinflammatory immune response and cytokine release [5].

Like the respiratory epithelial cells, endocrine organs also express the ACE2 receptor [6]. Individuals with SARS-CoV-2 infection are highly susceptible to disease-related metabolic dysregulation given the hyperinflammatory immune response of COVID-19 [7]. As a result, the precise pathophysiology of immunological dysregulation caused by COVID-19 infection remains unknown. However, a cytokine storm might occur from Type 1 T helper (Th1) cells' hyperinflammatory immunological response, which is followed by the activation of monocytes, macrophages, and neutrophils [5,8]. Interleukin-6 (IL-6) and tumor necrosis factor (TNF) are important cytokine components of the immune response generated by inflammatory cells, which are mostly attributable to a major change in metabolic regulation [5,9]. For instance, damage to the exocrine and endocrine pancreatic cells caused by the release of proinflammatory cytokines may lead to pancreatic insufficiency, and diabetes in severe COVID-19 patients [7,10].

This bidirectional relationship between COVID-19 infection and diabetes offers significant health risks to patients, as hyperglycemia may aggravate the hyperinflammatory immune response and tissue damage [11-13]. Adrenal insufficiency may also be induced by a severe COVID-19 infection, which results in decreased cortisol output and hormonal imbalances. These hormonal changes, particularly decreased cortisol levels, are involved in multiple steps in adipogenesis and lipolysis. However, minimal data is available on the effects of COVID-19 infection on adipose tissue function and regional body fat distribution.

This study mainly aims to further explore on the significant relations of COVID-19 infection with body fat distribution, changes in serum insulin, and homeostasis model assessment-estimated insulin resistance (HOMA-IR) levels before and after the infection.

## Materials and methods

Population

In this follow-up study, from July 2021 to September 2021, persons referred to a university-affiliated nutrition counselling clinic, Dietary Health Clinic, Tehran, Iran, were randomly selected for the study samples. At the start of the trial, all participants were informed about the study's objectives and possible benefits. Patients with metabolic problems (such as cancer, diabetes, liver disease, and thyroid abnormalities) were excluded, as were those on a specific diet or using chemotherapy, steroids, diabetes, or lipid-lowering medications. Furthermore, subjects who reported a history of severe COVID-19 infection and hospitalization due to COVID-19 were excluded. All participants were given a monthly balanced diet for weight control.

Questionnaire

A validated food frequency questionnaire (FFQ) with 168 food items was completed [14]. The questionnaire contained information on portion sizes for standard food sizes, in addition to food items. The questionnaire was filled out based on the frequency of specific foods consumed the previous day, month, and year and the standard portion size. The obtained values were converted to grams per day using a home scale manual. After that, each subject's daily total calorie, carbohydrate, protein, and fat consumption was calculated and reported. To compute each person's activity level, each activity was multiplied by the number of implementation days and duration of the activity, as determined by the metabolic equivalent (MET) unit. Anthropometric indices (height, weight, BMI) were measured by a trained specialist using standard tools including a tape meter, stadiometer (0.1 cm precision), and a 767-digital scale (± 0.1 kg; Seca GmbH & Co., Hamburg, Germany). BMI was obtained by dividing weight (kg) by the square of the height (m^2^). 

Measurements

Following a 12-hour fast, the participants' metabolic parameters were assessed using a 10 ml venous blood sample, and then the biochemical parameters were examined. The blood samples were immediately delivered to the laboratory, where blood serum was collected and used to calculate the variables after centrifugation at 2500 rpm for six to eight minutes. The insulin concentration was tested using commercial enzyme-linked immunosorbent assay (ELISA) kits, while the glucose level was determined using an enzymatic approach with a Pars Azmoon commercial kit (Pars Azmun Co., Tehran, Iran). The homeostatic model assessment (HOMA) was also used to evaluate insulin resistance using the HOMA Calculator v2.2.2 [15].

Body composition

There are several ways of determining body composition, including the dual-energy X-ray absorptiometry (DXA) technique, which was suggested for determining the fat mass (FM) and fat-free mass (FFM) [16,17]. DXA uses photon absorption in tissues to calculate body composition and reports total body weight in FM, FFM, and bone minerals. Lunar equipment (Lunar iDXA, GE HealthCare, Chicago, Illinois, United States) was used to examine body composition in this study, which employs DXA scanning to analyze the full body and is a standard instrument for body mass assessment [18]. This gadget measured fat mass and fat-free mass in the right and left arms, left and right thighs, trunk, and total fat and total fat-free mass, all reported in grams. According to the manufacturer's recommendations, this device scanned the individuals in the supine position for 15 minutes with no extra movement.

Follow-up

A follow-up session was scheduled for each participant one month after their first visit. In the follow-up session, questionnaires such as FFQ and physical activity were questioned for the second time. Following a 12-hour fast, a 10 ml venous blood sample was obtained and submitted to the lab for metabolic profiles such as glucose and insulin. The same procedure as the initial visit was used to analyze body composition and anthropometric indices one month later. The participants were given the instructions to refer to the clinic for re-measurements in case of a positive polymerase chain reaction (PCR) test of SARS-CoV-2 after a two-week quarantine from the first day of symptoms. In this regard, all follow-ups were done between 30 to 45 days. Those who reported mild to moderate COVID-19 infection (without hospitalization) on the second visit were selected as the case group and the healthy (non-infected) individuals as the control group.

Statistics

All data presented as mean values (± SD) were used for quantitative variables. The comparison between before and after COVID-19 infection was made using a paired t-test. All analyses were done in IBM SPSS Statistics for Windows, Version 20.0 (Released 2011; IBM Corp., Armonk, New York, United States) software, and a P-value of less than 0.05 was considered significant.

Ethical consideration

The entire method was in total conformity with the Helsinki Declaration. All people who took part in the study gave their written informed consent. The ethics committee of Fasa University of Medical Sciences approved the study (Approval number: IR.FUMS.REC.1400.138).

## Results

In a total of 441 participants, the mean age was 38.82±4.63 years. There were 224 (50.79%) male subjects and 217 (49.20%) were females. The participants were divided into two groups: the case group (n=221, 50.11%) and the control group (n=220, 49.89%). The case group consisted of 124 (56.1%) male and 97 (43.9%) female patients. There were 120 (54.55%) male and 100 (45.46%) female subjects in the control group.

Effects of SARS-CoV-2 on FM, FFM, and body weight

In the case group, the mean of TF before and after COVID-19 infection in men was 21.09±2.95 kg and 22.23±2.35 kg, respectively. After infection with COVID-19, the increase in TF composition was statistically significant (P-value=0.001). In addition, the mean TF in women was 17.612.03 kg and 18.092.4 kg, respectively, across the two periods. Although TF was greater in the female group after infection than before, the difference was negligible (P-value=0.137). Also, TF% showed a statistically significant difference between both men and women in the case group (P-value < 0.001). The mean of TF% in the male patients of the case group increased from 32.03±2.27 to 34.2±3.1 (Table 1). Also, the mean of TF% in female patients in the case group increased from 26.01±2.11 to 28.05±2.28. Interestingly, in the control group, TF% in both males and females was lower at follow-up. The mean of TF% in male participants in the control group decreased from 33.11±3.11 to 31.31±3.49, and that of female participants in the control group decreased from 26.58±2 to 24.08±2.77. In the control group (both males and females), the difference in TF% at follow-up was statistically significant (P-value <0.001). Also, when the changes in the variables were compared, the loss of FFM and body weight in subjects with COVID-19 was the same as the loss of FFM and body weight in subjects without COVID-19 (P-value > 0.05), whereas the changes in TF in kg and % between the two groups were significantly different (P-value < 0.001). More details about arm, legs, and trunk FM have been reported in Table. 1.

**Table 1:** Changes in body composition in two groups * represents the significance of P-value (of paired t-test) between baseline values and follow-up values COVID-19: coronavirus disease 2019

	Variable	Control			COVID-19		
		Baseline	Follow-up	Mean Difference P-value	Baseline	Follow-up	Mean Difference P-value
Male	Arm Fat (kg)	4.06±0.82	3±0.88	1.05±1.27 P-value= 0.000	3.98±0.67	2.79±1.04	1.19±1.28 P-value= 0.000
	Leg Fat (kg)	9.07±1.12	7.88±1.26	1.19±1.62 P-value= 0.000	9.22±1.01	8.13±1.35	1.1±1.67 P-value= 0.000
	Trunk Fat (kg)	15.96±2.71	16.2±3.23	-0.24±3.89 P-value= 0.503	17.05±2.62*	18.2±3.24*	-1.14±4.13 P-value= 0.003
	Arm Fat-Free (kg)	6.93±1.23	5.58±1.63	1.35±1.98 P-value= 0.000	7.05±1.26	5.68±1.6	1.37±2.15 P-value= 0.000
	Leg Fat-Free (kg)	12.34±2.28	10.63±2.64	1.71±3.38 P-value= 0.000	12.2±2.37	10.8±2.71	1.4±3.74 P-value= 0.000
	Trunk Fat-free (kg)	27.32±3.96	24.85±4.92	2.47±6.16 P-value= 0.000	26.88±4.53	24.3±5.15	2.59±6.99 P-value= 0.000
	Total Fat-free (kg)	46.59±5.04	41.05±5.84	5.35±8.20 P-value= 0.000	46.13±5.53	40.77±6.03	5.53±7.49 P-value= 0.000
	Total Fat (kg)	21.02±3.39	20.03±2.91	0.99±4.44 P-value= 0.016	21.09±2.95	22.23±2.35*	-1.14±3.85* P-value= 0.001
	Total Fat %	33.11±3.11	31.31±3.49	1.81±4.74 P-value= 0.000	32.03±2.27*	34.2±3.1*	-2.17±4.1* P-value= 0.000
Female	Arm Fat (kg)	3.98±0.85	2.88±1.01	1.1±1.31 P-value= 0.000	4.01±0.8	3.01±1.1	1±0.3 P-value= 0.000
	Leg Fat (kg)	5.81±1.18	4.51±1.22	1.3±1.78 P-value= 0.000	5.9±1.09	4.79±1.19	1.11±1.73 P-value= 0.000
	Trunk Fat (kg)	17.93±3.6	15.88±3.25	2.05±4.79 P-value= 0.000	17.77±2.84	18.36±2.87*	-0.6±3.94* P-value= 0.139
	Arm Fat-Free (kg)	6.99±1.27	5.42±1.71	1.57±2.09 P-value= 0.000	6.92±1.28	5.46±1.45	1.46±1.84 P-value= 0.000
	Leg Fat-Free (kg)	12.22±2.43	10.93±3.03	1.28±3.95 P-value= 0.002	12.45±2.37	11.46±3.07	0.99±3.69 P-value= 0.009
	Trunk Fat-free (kg)	26.78±3.34	24.74±5.38	2.04±5.79 P-value= 0.001	26.58±4.55	23.21±5.91	3.37±7.65 P-value= 0.000
	Total Fat-free (kg)	45.98±4.57	41.09±6.37	4.89±7.31 P-value= 0.000	45.94±5.39	40.13±6.92	5.81±8.86 P-value= 0.000
	Total Fat (kg)	18.18±2.89	17.26±3.12	0.92±4.44 P-value= 0.042	17.61±2.03	18.09±2.4*	-0.48±3.14* P-value= 0.137
	Total Fat %	26.58±2	24.08±2.77	2.51±3.28 P-value= 0.000	26.01±2.11	28.05±2.28*	-2.04±3.09* P-value= 0.000

**Figure 1:**
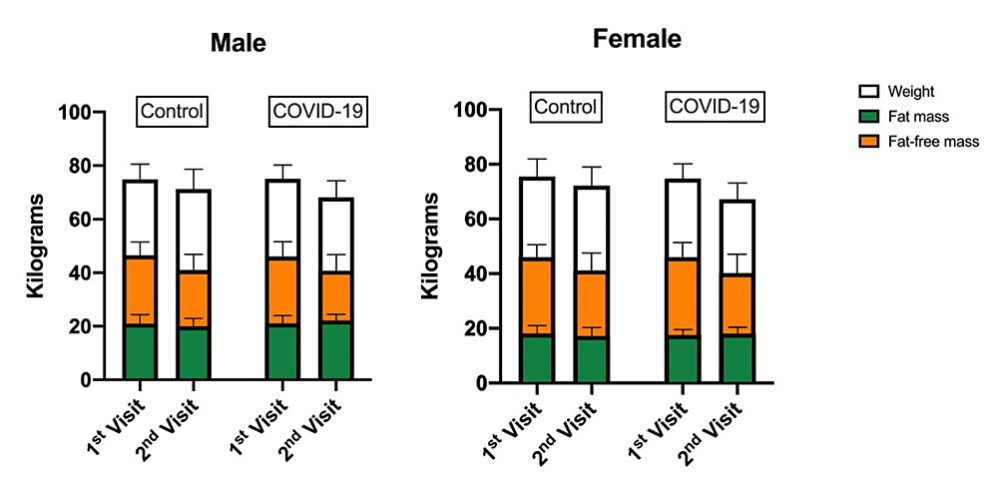
The mean of weight, total fat, and fat-free mass in kilograms on the first and second visit in COVID-19 (case) group and control group COVID-19: coronavirus disease 2019

The reduction in mean BMI in the case group (both male and female) was higher than in the control group. The mean BMI reduction in the case group before and after COVID-19 infection was 2.64±3.97 in males and 3.36±3.46 in females. Furthermore, the mean BMI reduction in the control group was 1.7±3.82 in males and 1.78±3.87 in females. When it came to weight loss during the COVID-19 pandemic, the case group individuals lost more weight than the controls. As a result, the average weight loss after SARS-CoV-2 infection was 6.95 ±7.8 in males and 7.56 ±8.1 in females, which was statistically significant (P-value < 0.001). Also, the control group showed decreased weight with a mean of 3.66±8.76 in males and 3.35±9.34 in females (P-value < 0.001). A unique finding of this study was the increase in TF% of COVID-19 subjects while there was a decrease in subjects' weight, interestingly, which may be due to loss of FFM (Figure 1).

Effects of SARS-CoV-2 on blood glucose, insulin level, and HOMA-IR

Table 2 shows further information on both groups' physical activity levels, total daily calorie consumption, and carbohydrate, fat, and protein intake. Furthermore, across both genders, both case and control groups demonstrated a statistically significant reduction in daily calorie consumption (P-value < 0.001). In addition, re-measurements revealed a statistically significant decline in physical activity in all individuals (P-value < 0.001).

**Table 2:** Changes in metabolic indices and food intake in two groups *represents the significance of P-value (of paired t-test) between baseline values and follow-up values COVID-19: coronavirus disease 2019

	Variable	Control			COVID-19		
		Baseline	Follow-up	Mean Difference P-value	Baseline	Follow-up	Mean Difference P-value
Male	Physical Activity (MET)	31.37±2.95	25.73±2.66	5.64±4.11 P-value= 0.000	30.84±2.93	25.81±3.03	5.03±4.37 P-value= 0.000
	Weight (kg)	74.85±5.73	71.18±7.48	3.66±8.76 P-value= 0.000	75.08±5.13	68.13±6.16*	6.95±7.8 * P-value= 0.000
	BMI	27.51±3.06	25.81±2.45	1.7±3.82 P-value= 0.000	27.88±2.83	25.23±2.34	2.64±3.97 P-value= 0.000
	Glucose (mg/dL)	100.65±7.56	102.55±8.6	-1.9±11.51 P-value= 0.073	101.34±7.87	111.55±7.51*	-10.21±10.68 * P-value= 0.000
	Insulin (μU/mL)	7.09±1.83	6.9±3.09	0.19±3.5 P-value= 0.557	6.86±1.88	11.03±3.05*	-4.17±3.65 * P-value= 0.000
	HOMA-IR	0.91±0.3	0.8±0.21	0.11±0.36 P-value= 0.001	0.92±0.3	1.23±0.28*	-0.31±0.4 * P-value= 0.000
	Energy intake (kcal)	2209.4±151.91	1892.08±206.98	317.32±264.58 P-value= 0.000	2188.49±179.57	1901.28±203.82	287.21±259.64 P-value= 0.000
	Carbohydrate intake (g)	300.85±20.55	266.67±21.3	34.17±27.23 P-value= 0.000	300.7±18.4	266.14±19.86	34.56±27.57 P-value= 0.000
	Fat intake (g)	84.14±9.15	77.66±11.41	6.48±14.69 P-value= 0.000	84.62±8.46	78.15±9.74	6.47±12.69 P-value= 0.000
	Protein intake (g)	84.81±10.16	84.81±10.16	4.01±14.5 P-value= 0.003	90.02±9.76	83.67±9.52	6.35±14.64 P-value= 0.000
Female	Physical Activity (MET)	32.88±2.97	30.29±3.32	2.59±4.04 P-value= 0.000	33.25±2.94	27.68±3.05*	5.57±3.99 * P-value= 0.000
	Weight (kg)	75.54±6.45	72.19±6.88	3.35±9.34 P-value= 0.001	74.78±5.44	67.22±5.95*	7.56±8.1 * P-value= 0.000
	BMI	27.81±2.98	26.03±2.47	1.78±3.87 P-value= 0.000	28.38±2.81	25.02±2.15*	3.36±3.46 * P-value= 0.000
	Glucose (mg/dL)	100.4±7.73	101.6±8.53	-1.2±11.46 P-value= 0.298	100.39±7.79	110.47±6.64*	-10.07±9.24 * P-value= 0.000
	Insulin (μU/mL)	6.73±1.93	6.79±2.97	-0.06±3.72 P-value= 0.871	6.98±1.91	10.35±2.75*	-3.37±3.49 * P-value= 0.000
	HOMA-IR	0.85±0.28	0.79±0.2	0.06±0.36 P-value= 0.090	0.91±0.25	1.23±0.33*	-0.32±0.41 * P-value= 0.000
	Energy intake (kcal)	2213.58±194.15	1890.22±206.24	323.35±289.26 P-value= 0.000	2198.43±162.92	1892.08±209.14	306.35±236.35 P-value= 0.000
	Carbohydrate intake (g)	301.02±17.84	269.09±17.87	31.93±24.76 P-value= 0.000	304.92±22.3	267.32±19.78	37.6±28.64 P-value= 0.000
	Fat intake (g)	84.42±8.54	79.12±10.56	5.29±14.79 P-value= 0.001	84.78±8.81	77.04±10.42	7.74±13.7 P-value= 0.000
	Protein intake (g)	90.62±11.09	84.13±7.72	6.49±12.5 P-value= 0.000	90.99±9.54	83.9±8.42	7.09±12.43 P-value= 0.000

**Figure 2:**
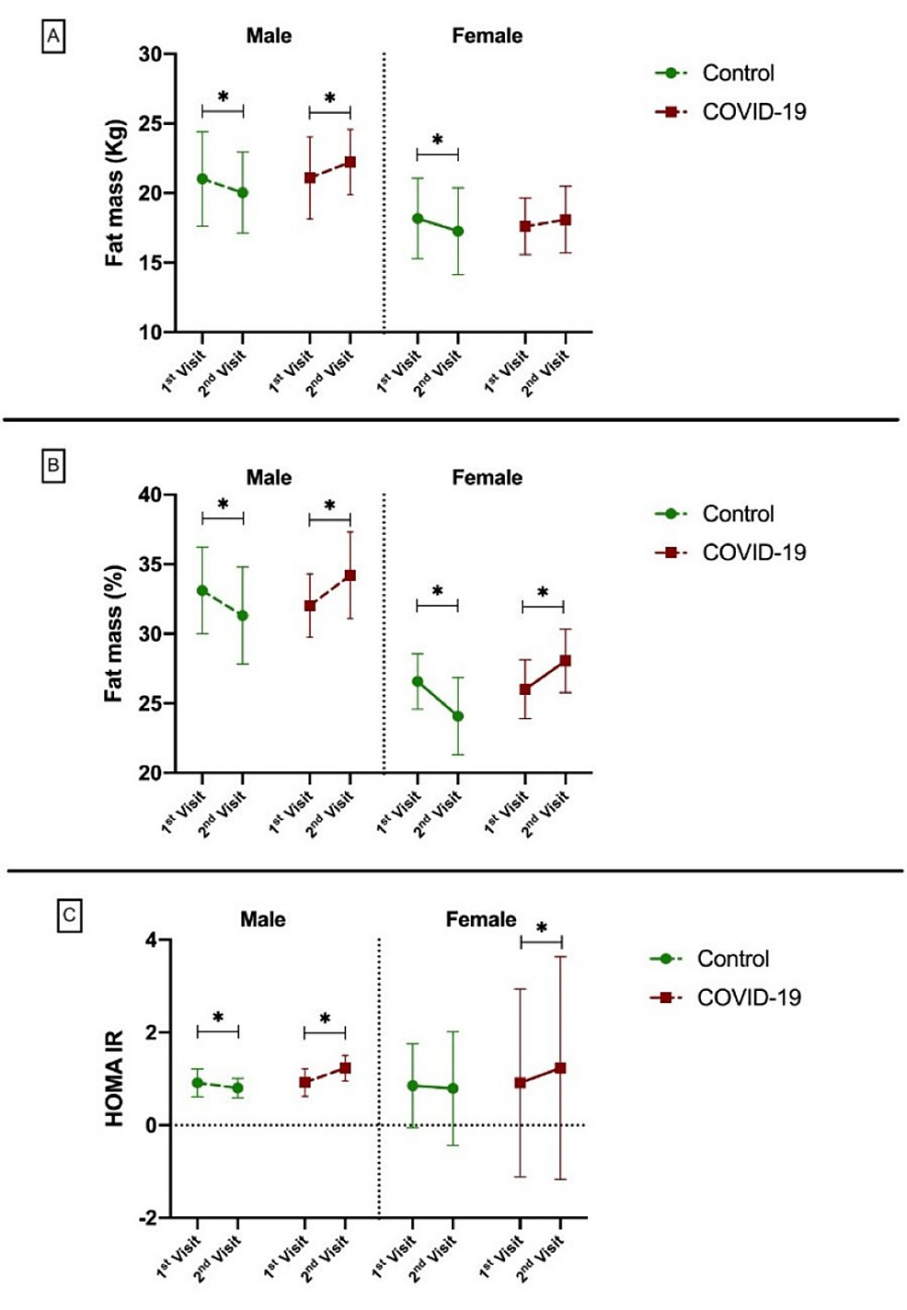
The mean of total fat (kg and percentage) and HOMA-IR on the first and second visit in COVID-19 and control groups in both sexes HOMA-IR: homeostasis model assessment-estimated insulin resistance; COVID-19: coronavirus disease 2019

In terms of COVID-19 effects on blood glucose, both males and females in the case group showed a statistically significant increase in mean glucose levels after the infection (P-value < 0.001). As presented in Table 2, the mean glucose level increased from 101.34±7.87 to 111.55±7.51 in males and 100.39±7.79 to 110.47±6.64 in females. However, both genders revealed similar glucose levels in the control groups. The same findings were achieved in terms of blood insulin levels in the case group (both males and females), which demonstrated an increase in insulin production following infection with COVID-19, which was statistically significant (P-value < 0.001). The controls, however, showed similar insulin levels. In the case group, the mean of homeostatic model assessment HOMA-IR in males was 0.92±0.3 and 1.23±0.28, and in females, it was 0.91±0.25 and 1.23±0.33, respectively, before and after COVID-19 infection. The difference between before and after COVID-19 in the case group (both males and females) was statistically significant (P-value < 0.001). However, the control group showed no differences in HOMA-IR levels in female participants (P-value > 0.05) (Table 2). Furthermore, when the changes in the variables were evaluated, the weight loss in participants with COVID-19 was significantly larger than the weight loss in those without COVID-19 (P-value < 0.001). Figure 2 depicts the mean TF in kg, percentage, and HOMA-IR differences in groups of both sexes before and after.

Moreover, the results of the correlation analysis between changes in the variables in both groups were provided in the Appendices. According to the results, the reduction of weight was correlated with the increasing levels of glucose and changes in HOMA-IR. Furthermore, arm FM was positively associated with the changes in HOMA-IR in subjects. Finally, TF% changes were correlated with the changes in glucose, insulin, and HOMA-IR.

## Discussion

This study evaluated subjects with and without COVID-19 after a monthly diet for weight control. The TF % decreased in participants without COVID-19 infection, as predicted, due to the low-calorie diet administered to both groups and the drop-in energy consumption of both groups (Table 2). Nonetheless, despite decreasing weight and consuming less calories, the TF% in COVID-19 patients was significantly greater after infection compared to their initial visit. As shown in Table 1, the amount of total body fat in the control group shows a decrease in both males and females, which the reduction of their energy intake can explain. Still, at the same time, in the case group, in both males and females, we observed an increase in total body fat while their energy intake and weight had decreased; the mean difference of changes between the two groups was also significant. Similar changes were observed for body fat percentage. As a result, we noticed a reduction in body fat percentage of roughly 2% in the control group (2.5% in females and 1.8% in males) and a 2% rise in body fat in the case group (2% in females and 2.17% in males). In terms of FFM in the arms, legs, and trunk, all participants (both case and control groups) saw significant reductions in their second visit compared to their first. Fasting blood glucose, insulin, and insulin resistance levels were significantly increased in the case group compared to their baselines. Moreover, our study suggested that the changes in TF% is correlated with the changes in metabolic indices such as glucose, insulin, and HOMA-IR changes.

This is the first study worldwide that evaluates the subjects' body composition before and after COVID-19 infection. Alongside the body composition, evaluating variables, such as energy intake, physical activity, weight, BMI, fasting blood glucose, insulin, and insulin resistance levels' repeated measurements make this study unique.

Previous studies have looked at the effect of body composition on COVID-19 infection. A study showed the predictive significance of obesity and body composition for SARS-CoV-2-infected individuals [19]. Furthermore, some studies have shown substantial links between body composition on axial CT slices and COVID-19 patients' clinical outcomes [20,21]. These studies go along with observational findings, which revealed that obesity is associated with an increased risk of severe COVID-19 disease [22,23]. Although many studies are evaluating the role of obesity and body composition in COVID-19 infection, to our knowledge, there are no studies evaluating the role of COVID-19 infection on body composition.

Previously, Gualtieri et al. studied the FM percentage derived by CT scans in COVID-19 patients admitted to the ICU and reported that the FM percentage had decreased after a 20-day interval [24]. These results contrast with our results, which may be due to their much smaller sample size, different population, and patients admitted in the ICU. Muscle loss and cachexia after COVID-19 have been reported previously [24-26]. Muscle atrophy occurs swiftly, according to previous research, with symptoms emerging within two days of inactivity. Muscle mass is reduced by roughly 6% after 10 days, and by around 10% after 30 days [25]. After at least a 30-day gap, males’ lean mass of arms, legs, and trunk decreased by 19.4%, 11.4%, and 9.6%, respectively, in our study. Females' lean mass decreased by 21%, 7.9%, and 12.6%, respectively. Regarding weight, we have observed a 9-10% decrease after COVID-19 infection compared to the 4-5% weight loss in the control group. This loss of muscle mass is linked to fiber denervation, neuromuscular junction injury, and increased protein breakdown, although a decrease in muscle protein synthesis mainly causes it. Inactivity has an impact on glucose homeostasis, particularly in muscles. Furthermore, from the cardiovascular system to skeletal muscle oxidative activity, aerobic capacity is lowered at all stages of the O_2_ cascade. Furthermore, during periods of physical inactivity, changes in energy balance are associated with fat deposition, systemic inflammation, and antioxidant defense activation, all of which accelerate muscle loss [26]. In this study, the COVID-19-positive group showed a more than 2% rise in fat deposition in both genders, while the control group showed a reduction in fat proportion in both genders.

Other findings of this study were changes in the glucose, insulin, and insulin resistance levels after COVID-19 infection in the case group. These variables experienced a significant increase after COVID-19 infection. Accordingly, there was no significant change in all three variables of fasting glucose, fasting insulin, and HOMA-IR in the control group. However, there was a significant difference in these variables in both genders of the case group. Previous papers in this field have repeatedly indicated these conclusions [27-30]. On admission, three and six months after discharge, Chen et al. examined insulin sensitivity and fasting insulin secretion in COVID-19 patients without diabetes, and presented the first evidence that COVID-19 may increase the risk of insulin resistance in people without diabetes [25,30]. In the case of fasting glucose levels, a study on hospitalized subjects in China suggested that elevated fasting blood glucose in the first week of hospitalization was associated with progression to severe illness of COVID‐19 [10]. Although many studies evaluated the effect of glucose and insulin level on COVID-19 outcome or severity, there are very limited studies on the effect of the glucose/insulin levels before and after COVID-19 infection like our study, which makes our findings in this regard unique.

The large sample size (n=441) was one of the study's strengths. The presence of a control group also adds to the study's credibility. Also, evaluating a variety of factors including body composition, caloric consumption, physical activity, weight, BMI, fasting blood glucose, insulin, and insulin resistance twice in a month's time, allows to interpret the body composition results alongside the weight, BMI, diet (energy intake), glucose, and insulin levels. Finally, this study is the first one regarding COVID-19 infection and its possible effect on the body composition of subjects.

This research has limitations, much like previous studies. To suggest a cause-and-effect relationship, close observation must be made at longer follow-up intervals, which can add to the importance of the research. Also, the current study was based on the individuals’ self-reported data in the case of FFQ and, therefore, the data were subjective and may be prone to substantial recall bias. Another disadvantage of the present study was that it only looked at an urban population; it is proposed that a similar study be conducted in a rural community to see if there are any possible differences and to generalize the results of this study.

## Conclusions

Interestingly, despite losing weight and consuming fewer calories, COVID-19 subjects' TF% increased considerably (more than 2%) after infection compared to their first visit. Participants who were not infected with COVID-19 had a lower TF%, as predicted. Individuals' total FFM decreased significantly on their second visit as compared to their first. The COVID-19 patients showed considerably greater fasting blood glucose, insulin, and insulin resistance levels than their controls. More studies should be performed in the future to find out the possible mechanism of increasing TF% and decreased FFM in COVID-19 subjects. Moreover, individuals with COVID-19 infection may require tailored medical nutrition therapy to improve short and long-term COVID-19 outcomes such as muscle loss and fat accommodation.

## Figures and Tables

**Figure 3:**
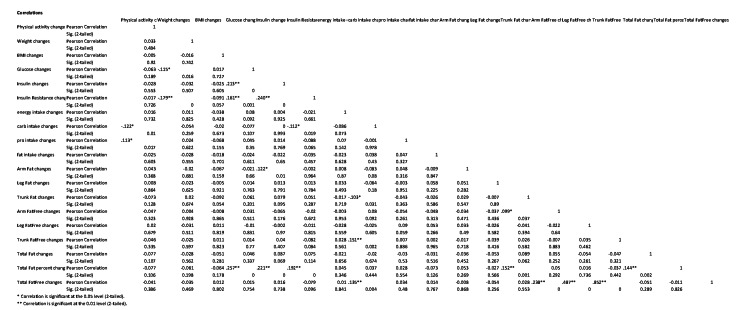
Correlation between body composition variables and metabolic variables.
